# Factors Associated with Uncontrolled Hypertension among Renal Transplant Recipients Attending Nephrology Clinics in Nairobi, Kenya

**DOI:** 10.1155/2015/746563

**Published:** 2015-07-14

**Authors:** Mary N. Kubo, Joshua K. Kayima, Anthony J. Were, Seth O. McLigeyo, Elijah N. Ogola

**Affiliations:** ^1^Department of Clinical Medicine and Therapeutics, University of Nairobi (UoN), P.O. Box 60337-00200, Nairobi, Kenya; ^2^Kenyatta National Hospital (KNH), Nairobi, Kenya

## Abstract

*Objective. *To determine the factors associated with poor blood pressure control among renal transplant recipients in a resource-limited setting.* Methods*. A cross-sectional study was carried out on renal transplant recipients at the Kenyatta National Hospital. Sociodemographic details, blood pressure, urine albumin : creatinine ratio, and adherence using the MMAS-8 questionnaire were noted. Independent factors associated with uncontrolled hypertension were determined using logistic regression analysis.* Results*. 85 subjects were evaluated. Mean age was 42.4 (SD ± 12.2) years, with a male : female ratio of 1.9 : 1. Fifty-five patients (64.7%) had uncontrolled hypertension (BP ≥ 130/80 mmHg). On univariate analysis, male sex (OR 3.7, 95% CI 1.4–9.5, *p* = 0.006), higher levels of proteinuria (*p* = 0.042), and nonadherence to antihypertensives (OR 18, 95% CI 5.2–65.7, *p* < 0.001) were associated with uncontrolled hypertension. On logistic regression analysis, male sex (adjusted OR 4.6, 95% CI 1.1–19.0, *p* = 0.034) and nonadherence (adjusted OR 33.8, 95% CI 8.6–73.0, *p* < 0.001) were independently associated with uncontrolled hypertension.* Conclusion*. Factors associated with poor blood pressure control in this cohort were male sex and nonadherence to antihypertensives. Emphasis on adherence to antihypertensive therapy must be pursued within this population.

## 1. Background

Renal transplantation is the preferred therapy for patients with end stage renal disease, with improved quality of life and survival compared to dialysis. The prevalence of hypertension remains high at 80–90% [1], despite improved glomerular filtration rate and fluid status after transplantation. The pathogenesis of posttransplant hypertension is multifactorial. It has been linked to both recipient and donor factors, including immunosuppressant use (particularly cyclosporine) [2], advancing donor age, transplant renal artery stenosis, and transplant dysfunction.

Treatment goals of hypertension among renal transplant recipients are more stringent than in the general hypertensive population. KDIGO (kidney disease improving global outcomes) guidelines recommend target blood pressure levels of <130/80 mmHg [3]. In the UK, up to 50% of renal transplant recipients were found to have uncontrolled hypertension [4]. In Kenya, the figures are much higher, with unpublished data showing that 79–84% of renal transplant recipients have not met target blood pressure levels.

The consequences of uncontrolled hypertension in this population include increased cardiovascular mortality and morbidity. Cardiovascular disease is the leading cause of mortality among renal transplant recipients, more than mortality from infection and malignancy combined [5]. In addition, poorly controlled hypertension is associated with reduced allograft survival, as shown in the Collaborative Transplant Study [6].

Nonadherence to antihypertensive treatment is an important and often unrecognized risk factor that contributes to reduced control of blood pressure. The WHO estimates that up to 50% of patients with chronic illnesses are nonadherent to long-term therapy [7].

Indeed, nonadherence to antihypertensive medications as determined by the 8-item Morisky Medication Adherence scale (MMAS-8) was found to be predictive of elevated systolic and diastolic pressures among patients in Portugal [8]. Renal transplant recipients may also have preferential adherence to their immunosuppressant therapy over nonimmunosuppressant therapy including antihypertensive medications. This was shown by Terebelo et al., who demonstrated adherence rates of 81.6% to immunosuppressant therapy versus 55.1% to nonimmunosuppressant therapy among renal transplant recipients in New York, USA (*p* = 0.028) [9]. Other predictors of uncontrolled hypertension among renal transplant recipients include older recipient and donor age, male sex, higher levels of proteinuria, and low GFR, as well as cyclosporine and steroid use [10–13].

Uncontrolled hypertension among our renal transplant recipients is common. We thus sought to determine demographic, social, and clinical factors associated with poor blood pressure control. We also sought to determine adherence rates to antihypertensive therapy, and its relation to blood pressure control.

## 2. Methods

### 2.1. Study Design

It is cross-sectional.

### 2.2. Setting

Kenyatta National Hospital (KNH) Renal Unit, Nairobi, Kenya, is a national referral and teaching hospital with a rapidly growing renal transplant program.

### 2.3. Participants

The entire cohort of renal transplant recipients were hypertensive, more than 2 months posttransplant, 18 years of age and above, and not on dialysis due to nonfunctional grafts and gave informed written consent.

### 2.4. Objective

To determine factors associated with poor blood pressure control among renal transplant recipients.

### 2.5. Definitions

Hypertension was defined as either the use of antihypertensive therapy* or* systolic blood pressure ≥140 mmHg and/or diastolic blood pressure ≥90 mmHg. Controlled blood pressure was defined as BP < 130/80 mmHg as per KDIGO guidelines [3].

Level of adherence to antihypertensive medications was categorized as high (score 8), medium (score 6-7), or low (score < 6). Medium and low adherence levels were both considered nonadherent [8].

### 2.6. Data Collection

Informed written consent was obtained prior to patient enrolment into the study. Participants' sociodemographic history and clinical history were then recorded in a predesigned questionnaire. Blood pressure measurement [14] and anthropometric measures [15] were taken using standard procedure with duly calibrated machines. In addition, blood pressure readings from the preceding two clinic visits were also recorded. An average of the three blood pressure readings was then calculated. 2 mls of blood for serum and 5 mls of urine were taken for measurement of serum creatinine and urine albumin : creatinine ratio, respectively. Adherence to antihypertensive therapy was assessed using the MMAS-8 questionnaire. This is an 8-item questionnaire with high sensitivity (93%) in determining adherence to antihypertensive therapy [16]. It has also undergone successful cross-cultural validation. Data was collected between November 2012 and February 2013.

### 2.7. Laboratory Methods

Serum creatinine was determined using the Mindray Clinical Chemistry Analyzer. Urine albumin : creatinine ratio measurement was done using the Clinitek Microalbumin Analyzer.

Daily internal quality control was carried out before sample analysis to validate the results obtained. External quality assessment was provided by Huqas company, a local proficiency testing provider.

### 2.8. Statistical Analysis

Statistical analysis was carried out using Statistical Package for Social Sciences (SPSS) version 17.0, (SPSS Inc., Chicago, IL, USA).

Association between uncontrolled hypertension and categorical variables (sex, adherence to antihypertensives, and cyclosporine use) was done using the Chi-square test of association or Fischer's exact test for small numbers, as appropriate. Student's *t*-test was used to compare means of continuous variables (level of proteinuria, age).

Estimates of the risks of uncontrolled hypertension were presented as odds ratios. The independent factors associated with uncontrolled hypertension were determined using logistic regression analysis. All the statistical tests were performed at 5% level of significance.

### 2.9. Ethical Issues

Ethical approval was given by the KNH/UoN Ethics and Research Committee, and all procedures were in accordance with the institutional ethical standards. Patients gave informed written consent. Confidentiality of patient records was maintained at all times.

## 3. Results

### 3.1. Baseline Characteristics

All 85 eligible renal transplant recipients who gave consent were enrolled. Baseline characteristics are described in Table 1. Average age was 42.2 (±12.2) years. Male : female ratio was 1.9 : 1. Mean duration after transplantation was 30.2 months, with a range between 3 and 233 months. Mean serum creatinine was 118.2 (±37.2) *μ*mol/L. Microalbuminuria was noted in 42.4% of patients, and 10.5% had macroalbuminuria. Mean BMI was 24.8 (±4.4) kg/m^2^. 55 patients (64.7%, 95% CI 56.4–79.7) had uncontrolled hypertension. Mean systolic blood pressure was 132.5 (±17.3) mmHg and mean diastolic blood pressure was 82.8 (±13.1) mmHg. Mean number of antihypertensive medications taken was 2.1 (±0.9).

### 3.2. Nonadherence to Antihypertensives

Two-thirds of the patients (67.1%) were nonadherent to antihypertensive medication. The mean MMAS-8 score was 6.8. Of the nonadherent patients, about a quarter (23.5%) forgot to take their medications.

### 3.3. Predictors of Uncontrolled Hypertension

In unadjusted univariate analysis, male sex (OR 3.7, 95% CI 1.4–9.5, *p* = 0.006), higher levels of proteinuria (*p* = 0.042), and nonadherence to antihypertensive medication (OR 18.0, 95% CI 5.2–65.7, *p* < 0.001) were associated with uncontrolled hypertension. Association between uncontrolled hypertension and other variables including number of antihypertensive medications, recipient and donor age, GFR, and cyclosporine use did not reach statistical significance (Table 2). On logistic regression analysis, independent predictors of uncontrolled hypertension were male sex (adjusted OR 4.6, 95% CI 1.1–19.0, *p* = 0.034) and nonadherence to antihypertensives (adjusted OR 33.8, 95% CI 8.6–73.0, *p* < 0.001) (Table 2).

## 4. Discussion

We found a high prevalence of uncontrolled hypertension among our renal transplant recipients. This is significant because cardiovascular disease remains the leading cause of mortality among renal transplant recipients [5]. Our purpose was to determine factors that may contribute to the poor blood pressure control in our recipients. Some of these factors may be modifiable, leading to better control and ultimately, improved patient and allograft survival.

The study population was relatively young, mean age of 42.4 (±12.2) years. This is about a decade younger than that noted in studies carried out in developed countries. Mason et al. found a mean age of 50.2 years among renal transplant recipients in UK [11]. Chronic kidney disease has been documented to occur at an earlier age in tropical countries [17].

Male : female ratio was 1.9 : 1. The male predominance is a reflection of the gender distribution of patients with chronic kidney disease attending nephrology clinics locally. Males may be more economically advantaged compared to their female counterparts, enabling them to access the relatively expensive renal transplantation services.

### 4.1. Blood Pressure Control

64.7% of our study participants did not achieve target blood pressure levels. This figure is higher than that found among renal transplant recipients in Jordan [10] and in UK [11] at 42% and 50%, respectively. Our higher rates of uncontrolled hypertension could be explained by the differences in the duration after transplantation in these populations. Mean duration after renal transplantation was much longer in UK [11] (76 months) and Jordan [10] (38 months) compared to ours at 30.2 months. Previous studies have demonstrated that blood pressure control improves as the duration after transplant increases [1]. This is due to stabilization of graft function with improved GFR, as well as tapering-off of immunosuppressant medication doses, especially cyclosporine and steroids that have been implicated in causation of hypertension.

### 4.2. Adherence to Antihypertensive Medications

Only a third of our study population was found to be fully adherent to their antihypertensive medications. Our adherence level was comparable to that found by Achieng' et al. in a study done among patients with hypertension attending medical outpatient clinics at the Kenyatta National Hospital [18]. 31.8% of her study population were adherent to therapy as assessed by the Hill-Bone questionnaire. About a quarter of our patients forgot to take their antihypertensive medications. This represents a possible area of intervention to improve adherence rates among our renal transplant recipient population. Other possible reasons for low levels of adherence include high cost of drugs [19, 20] as well as the high pill burden faced by renal transplant recipients.

### 4.3. Gender versus Blood Pressure Control

Male sex was independently associated with uncontrolled hypertension in our study. In an analysis of data from the third National Health and Nutrition Survey, Hyman and Pavlik found male sex to be independently associated with uncontrolled blood pressure among 16,095 adults in the US [21]. This sex difference could be due to increased plasma renin activity in males or androgen stimulation of sodium reabsorption and vasoconstrictor molecules such as endothelin [22]. In UK, female sex was associated with better blood pressure control among renal transplant recipients [11].

### 4.4. Nonadherence versus Blood Pressure Control

Nonadherence to antihypertensive medications was independently associated with poor blood pressure control among our study participants. Oliveira-Filho et al. in Portugal also demonstrated that nonadherence as measured by the same tool, the MMAS-8, was predictive of elevated systolic and diastolic pressures among patients with hypertension [8]. To our knowledge, there has been no other study looking at the association between adherence and blood pressure control specifically among renal transplant recipients, and we have no reason to believe that this population should be different from the general hypertensive population in this regard.

### 4.5. Strengths and Limitations

Our study had several strengths. It is, to our knowledge, the first study in Sub-Saharan Africa to explore the factors linked with uncontrolled hypertension among renal transplant recipients. Secondly, the tool used for assessment of adherence was the MMAS-8 questionnaire, validated for use in different cultural settings and with a higher reliability than its predecessor, the MMAS-4 (*a* = 0.83 versus 0.63) [16].

On the other hand, we faced several limitations. As the renal transplant population is still growing, we were limited in terms of participant numbers, as this is a limited sized cohort of patients. However, all eligible patients were included. In addition, this was a cross-sectional study, and hence causality cannot be inferred. Finally, consequences of the poor blood pressure control were not studied.

Nevertheless, our study was able to explore the possible factors that may be associated with uncontrolled hypertension in this unique population of patients. We were further able to determine the level of adherence to antihypertensive medications and possible reasons for nonadherence.

## 5. Conclusions

There is a high rate of uncontrolled hypertension among local renal transplant recipients. It was noted that adherence to antihypertensive medications was low. Predictors of poor blood pressure control were male sex and nonadherence to antihypertensive therapy.

We thus recommend intensification of blood pressure control among local renal transplant recipients. Studies to look into the patient-perceived reasons for nonadherence would be prudent. Strategies to improve levels of adherence to antihypertensive medications among renal transplant recipients should also be put in place.

Finally, we recommend follow-up analytical studies as the renal transplant recipient population grows, to look further into the associations alluded to in this cross-sectional study.

## Figures and Tables

**Table 1:** Baseline characteristics.

Variables	Frequency (%)
Age in years (recipient)	
Mean (SD, SEM)	42.4 (12.2, 1.7)
Min-Max	18–68

Sex (recipient)	
Male	56 (65.9**)**
Female	29 (34.1)

Age in years (donor)	
Mean (SD, SEM)	33.2 (8.5, 1.4)
Min-max	21–54

Health insurance	
Yes	85 (**100**)

Serum creatinine (*µ*mol/L)	
Mean (SD, SEM)	118.2 (37.2, 6.3)
Min-max	69–321

Urine albumin : creatinine ratio (mg/g)	
<30	40 (47.1)
30–300 (microalbuminuria)	36 (42.4)
>300 (macroalbuminuria)	9 (10.5)

Mean BMI in kg/m^2^ (SD, SEM)	24.8 (4.4, 0.7)

History of acute rejection	
Yes	14 (6.5)
No	71 (83.6)

**Table 2:** Association between uncontrolled hypertension and selected correlates.

Variable	Univariate analysis				Multivariate analysis	
	Hypertension		OR (95% CI)	*p* value	Adjusted OR (95% CI)	*p* value
	Uncontrolled (*n* = 55)	Controlled (*n* = 30)				
Age of recipient in years (SD)	43.3 (11.3)	40.7 (13.6)	—	0.339	—	

Sex of recipient						
Male	42 (76.4%)	14 (46.7%)	**3.7** (1.4–9.5)	**0.006**	4.6 (1.1–19.0)	**0.034**
Female	13 (23.6%)	16 (53.3%)	1.0		1.0	

Recipient's BMI in kg/m^2^ (SD)	24.5 (4.0)	25.3 (5.1)	—	0.382	—	

Age of donor in years (SD)	33.0 (8.4)	33.7 (8.7)	—	0.724	—	

GFR in mL/min/1.73 m^2^ (SD)	73.4 (20.4)	75.6 (14.2)	—	0.598	—	

Proteinuria in mg/g (IQR)	75.0 (27.0–150.0)	30.0 (20.0–80.0)	—	**0.042**	1.00 (0.96–1.03)	0.894

Rejection history						
Yes	10 (18.2%)	4 (13.3%)	1.4 (0.4–5.1)	0.762	—	
No	45 (81.8%)	26 (86.7%)	1.0			

Cyclosporine						
Yes	44 (80.0%)	21 (70.0%)	1.7 (0.6–4.8)	0.299	—	
No	11 (20.0%)	9 (30.0%)	1.0			

Cyclosporine dose mg/kg (SD)	2.8 (1.6)	2.9 (1.7)	—	0.950	—	

Adherence						
High	5 (9.1%)	23 (76.7%)	1.0		1.0	
Medium	28 (50.9%)	7 (23.3%)	18.0 (5.2–65.7)	**<0.001**	33.8 (8.6–73.0)	
Low	22 (40.0%)	0 (0.0%)	—	**<0.001**		**<0.001**

Number of antihypertensives	2.0 (2.0–3.0)	2.0 (1.0–3.0)	—	0.407	—	
